# Reducing mental health-related stigma among medical and nursing students in low- and middle-income countries: a systematic review

**DOI:** 10.1017/S2045796019000167

**Published:** 2019-04-01

**Authors:** E. Heim, C. Henderson, B. A. Kohrt, M. Koschorke, M. Milenova, G. Thornicroft

**Affiliations:** 1 Department of Psychology, University of Zurich, Switzerland; 2 Health Services and Population Research Department, Institute of Psychiatry, Psychology and Neuroscience, King’s College London, UK; 3 Department of Psychiatry, George Washington University, USA; 4 Centre for Global Mental Health, Institute of Psychiatry, Psychology and Neuroscience, King’s College London, UK

**Keywords:** Attitudes, mental health, mental illness stigma, quality of care, systematic reviews

## Abstract

**Aims:**

This systematic review compiled evidence on interventions to reduce mental health-related stigma among medical and nursing students in low- and middle-income countries (LMICs). Primary outcomes were stigmatising attitudes and discriminatory behaviours.

**Methods:**

Data collection included two strategies. First, previous systematic reviews were searched for studies that met the inclusion criteria of the current review. Second, a new search was done, covering the time since the previous reviews, i.e. January 2013 to May 2017. Five search concepts were combined in order to capture relevant literature: stigma, mental health, intervention, professional students in medicine and nursing, and LMICs. A qualitative analysis of all included full texts was done with the software MAXQDA. Full texts were analysed with regard to the content of interventions, didactic methods, mental disorders, cultural adaptation, type of outcome measure and primary outcomes. Furthermore, a methodological quality assessment was undertaken.

**Results:**

A total of nine studies from six countries (Brazil, China, Malaysia, Nigeria, Somaliland and Turkey) were included. All studies reported significant results in at least one outcome measure. However, from the available literature, it is difficult to draw conclusions on the most effective interventions. No meta-analysis could be calculated due to the large heterogeneity of intervention content, evaluation design and outcome measures. Studies with contact interventions (either face-to-face or video) demonstrated attitudinal change. There was a clear lack of studies focusing on discriminatory behaviours. Accordingly, training of specific communication and clinical skills was lacking in most studies, with the exception of one study that showed a positive effect of training interview skills on attitudes. Methods for cultural adaptation of interventions were rarely documented. The methodological quality of most studies was relatively low, with the exception of two studies.

**Conclusions:**

There is an increase in studies on anti-stigma interventions among professional students in LMICs. Some of these studies used contact interventions and showed positive effects. A stronger focus on clinical and communication skills and behaviour-related outcomes is needed in future studies.

## Introduction

In low- and middle-income countries (LMICs), the lack of financial and human resources to meet the needs of people with mental disorders has led to an alarming ‘treatment gap’, which in some countries is up to 90% (Degenhardt *et al*., 2017; Thornicroft *et al*., 2017; Alonso *et al*., 2018). To address this treatment gap, the World Health Organization (WHO) Mental Health Gap Action Programme (mhGAP) promotes the integration of mental health into primary health care (Funk *et al*., 2008). In this context, nurses and general practitioners increasingly provide first-contact care (i.e. detection and management) to people with mental health problems. Since they are not specialised in mental health, it is important to carefully examine and address mental health-related stigma in these two professional groups. Mental health-related stigma is defined as lack of knowledge about mental health, as well as negative attitudes and discriminatory behaviours towards people with mental disorders (Thornicroft *et al*., 2007).

Evidence shows that in most regions of the world, people with mental illness are confronted with mental health-related stigma (e.g. Thornicroft *et al*., 2009; Lasalvia *et al*., 2013). Mental health-related stigma is not limited to the general population but has been shown also to be prevalent among health professionals worldwide (Henderson *et al*., 2014; Vistorte *et al*., 2018), with negative consequences for people with mental disorders, such as limited access to health care (Thornicroft, 2008) and increased mortality (Thornicroft, 2011; Liu *et al*., 2017). Negative attitudes and discriminatory behaviours can be addressed in specific interventions with primary health care staff, but ideally, they are incorporated in health and mental health education from the beginning of professional training, i.e. among university students.

Professional students in medicine and nursing are the future workforce in primary health care, and they can be trained and sensitised for mental health-related stigma. Tackling students’ negative attitudes and unpleasant feelings (e.g. anxiety) related to people with mental disorders before their first contact with patients is crucial, in order to ensure that they make more positive experiences and develop interest and competence in mental health care. However, empirical evidence on how to reduce stigma among health care workers or professional students in general, and particularly in LMICs, is scarce (Yamaguchi *et al*., 2013; Henderson *et al*., 2014; Mehta *et al*., 2015). In a systematic review, Mehta *et al*. (2015) reviewed interventions to reduce mental health-related stigma among different populations. This review included four studies targeting medical students in LMICs. Another systematic review focused on university and college students and found 35 anti-stigma intervention studies (Yamaguchi *et al*., 2013). The authors identified only three studies from LMICs, and a lack of interventions with medical and nursing students in general. In their review, 66% of studies focused on college students, thus not specifically on professional students in the health sector. In this review, direct or video-based social contact interventions were the most effective in reducing stigmatising attitudes, a result that was also found in the general population (Mehta *et al*., 2015).

Aside from developing effective interventions for reducing mental health-related stigma among professional students, measuring outcomes of such interventions is an additional challenge. Most studies measure outcomes in terms of knowledge and attitudes, which is easier to measure than discriminatory behaviours (Henderson *et al*., 2014). This was confirmed in the previously mentioned systematic review by Yamaguchi *et al*. (2013), who found that outcomes of stigma interventions with professional students are most often measured in terms of knowledge and attitudes, such as desired social distance to people with mental disorders. Overall, little evidence exists on behavioural outcomes of anti-stigma interventions. This lack of evidence might be explained by the fact that behavioural outcomes are either measured by asking patients about their experiences with health professionals, or through observation of contact with patients, and such data are more difficult to gather than survey-based assessments of knowledge and attitudes. However, since discriminatory behaviours are the stigma-component that directly affects people with mental disorders during the interpersonal contact, a lack of evidence on how to improve such behaviours is an important shortcoming in literature.

The present study provides an update to the previously conducted systematic reviews on interventions to reduce mental health-related stigma among professional students, with a particular focus on professional students in LMICs. Since only three studies from LMICs were identified in a previous systematic review (Yamaguchi *et al*., 2013), and considering the primary focus of the WHO mhGAP on resource-scarce contexts, compiling evidence from LMICs is important. Moreover, if such interventions are implemented in culturally diverse contexts, it is particularly relevant to consider cultural aspects in anti-stigma interventions. Evidence on such cultural aspects has not been included in previous systematic reviews with professional students. Our narrative overview of interventions includes the content of trainings and didactic methods, the type of measures used to assess the outcomes of these interventions, and the methods applied for cultural adaptation of interventions and outcome measures (if any). The original search included professional students and health professionals, e.g. general practitioners, nurses, or lay health workers. Results on health professionals were presented in a separate paper (Heim *et al*., 2018), whereas the present paper focuses on interventions with professional students. The present paper aims to identify effective interventions to reduce mental health-related stigma among professional students in LMICs, as they are the future workforce in primary health care.

## Data collection, extraction and analysis

**Fig. 1.**
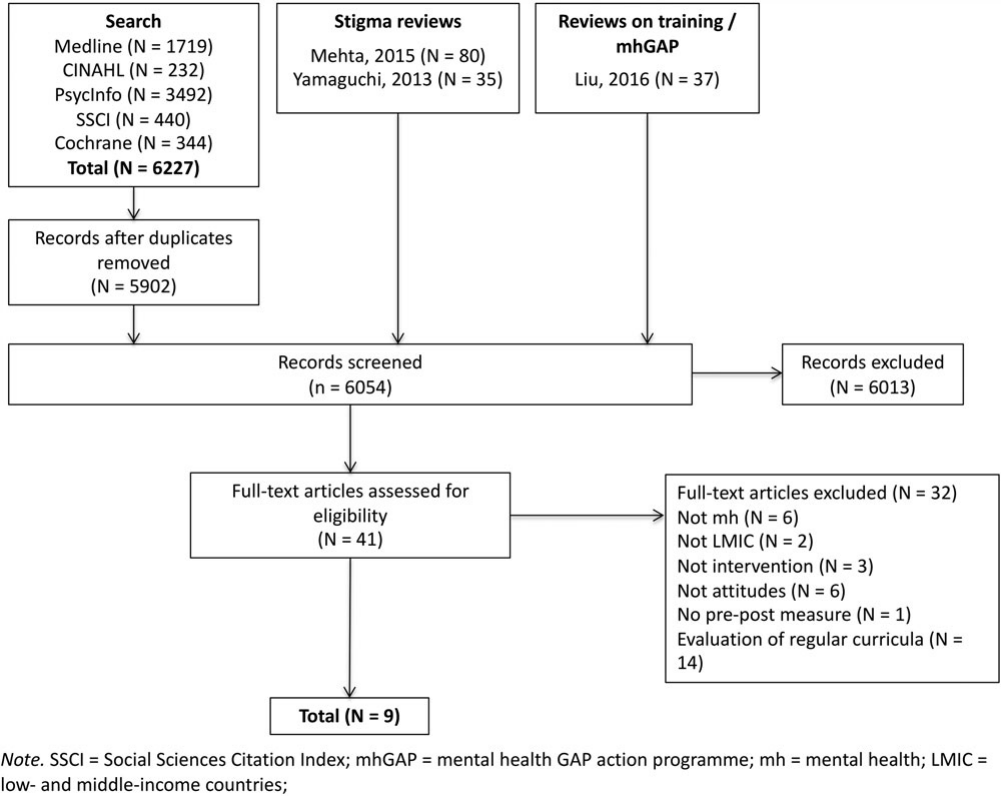
Preferred Reporting Items for Systematic Reviews and Meta-Analyses (PRISMA) diagram with a systematic search and selection process.

This study was listed in the PROSPERO register for systematic reviews (registration number CRD42017065436). Data collection included two different strategies. First, existing systematic reviews (Yamaguchi *et al*., 2013; Mehta *et al*., 2015; Liu *et al*., 2016) were searched for studies that met the inclusion criteria of the present review. Second, a new search was done, covering the time since the previous reviews, i.e. January 2013 to May 2017. The new search was run on 6 May 2017 and covered the following databases: PsycINFO, MEDLINE (Ovid), CINAHL, Social Sciences Citation Index and Cochrane (only Trials). Five search concepts were combined in order to capture relevant literature: stigma (e.g. stigma, discrimination, or stereotype); mental health (e.g. depression, anxiety, or schizophrenia); intervention (e.g. randomised controlled trial, evaluation, or pre-post); primary health care staff (e.g. general practitioners, health care workers, or professional students); and countries classified as LMICs by World Bank (2016) according to their gross national income. In the search, we included both country names (e.g. Afghanistan) and population adjectives (e.g. Afghan). Search terms within one concept were separated with OR, and the concepts were combined with AND. The search strategies were adapted from the previous reviews (Yamaguchi *et al*., 2013; Mehta *et al*., 2015; Liu *et al*., 2016). The complete search strategy (only Medline) can be accessed in the online Supplementary Material. The PRISMA diagram showing the data collection process can be found in Fig. 1. Of the 6054 titles and abstracts that were screened, 6013 were excluded because they were not related to the topic of the systematic review, did not address mental health-related stigma, were not interventions, did not address professional students, or were not conducted in LMICs. Reasons for exclusion are indicated for the full-text screening only.

Inclusion and exclusion criteria were defined along the participants, interventions, comparators and outcomes (PICO) approach. Participants in stigma interventions were professional students in medicine and nursing. Studies were included if they tested a specific anti-stigma intervention. Studies were excluded if they evaluated regular curricula because we were interested in specific interventions that can be added to such curricula to specifically address mental health-related stigma. We also excluded studies that trained or evaluated knowledge and behaviour/skills only, without an attitudinal (stigma-related) component, since our main focus was how to change attitudes and discriminatory behaviours rather than knowledge only. We also excluded studies that did not include a pre-training assessment to ensure a minimal quality of included studies. Both qualitative and quantitative studies were included, and no comparator was defined.

For a detailed content analysis, included full texts were introduced to the qualitative data analysis software MAXQDA (version 12.3.3). A coding system was developed including the following categories: stigma intervention content (e.g. theory, diagnostic skills, relationship skills), didactic methods (e.g. lecture, role plays, contact with patients), the mental disorder (e.g. depression, psychosis) the intervention focused on, whether the anti-stigma intervention was culturally adapted, the type of outcome measure (e.g. validated or non-validated questionnaire, qualitative interviews, behavioural observation), and primary outcomes. The analysis of outcomes focused only on attitudes and behaviour, not on knowledge. Outcome measures regarding knowledge are very diverse and would provide results that are difficult to compare, and knowledge was not the main focus of the present review (see inclusions criteria). Two raters coded the full texts independently and discussed possible differences until finding an agreement. Additionally, we critically appraised the methodological quality of the included studies along the following four criteria: whether the study implemented a control group, whether group allocation was randomised, how the random sequence was generated, and the handling of incomplete data. None of the studies used statistical measures for handling missing data. We assessed whether the percentage of missing data at post-assessment was below 5%. The quality of a study was rated as high if a study fulfilled the four criteria, moderate if three criteria were fulfilled, and low if it fulfilled two or less criteria.

A meta-analysis was originally planned but could not be calculated for the following reasons: of the six studies that had used a control group, two studies compared two different didactic methods, and one study had administered an outcome measure with a binary response format (Altindag *et al*., 2006).

## Results

### Settings and populations

A total of *K* = 9 studies were included in the analysis (see Table 1). They were from six different LMICs, with three studies from Turkey and two from Brazil. Participants were medical students (*k* = 5), nursing students (*k* = 3), or both (*k* = 1). Sample sizes ranged from 44 to 205, with five studies having sample sizes of *N* > 100. Four studies covered mental disorders in general, two studies addressed stigma related to schizophrenia, two studies abuse of alcohol, and one study addressed depression.

**Table 1.** Summary of included studies describing training with professional students

Reference	Country	N	Intervention	Design	Measure(s)	Main results (Descriptive analysis)
Altindag *et al*. (2006)	Turkey	60	Anti-stigma program for first-year medical students	Controlled design with pre-, post- and 1-month follow-up Control group: No training	32-item questionnaire designed for rating the attitudes towards schizophrenia, used in different studies in Turkey.	Statistically significant change in one out of seven items on social distance between pre-assessment and follow-up (i.e. ‘people with schizophrenia are dangerous’).
de Vargas (2013)	Brazil	195	Clinical practicum for nursing students in a service specialised in the treatment of abuse of alcohol	Quasi-experimental controlled pre-post design, random allocation	Attitude Scale Towards Alcohol, Alcoholism and Alcoholics (EAFAAA, Vargas and Villar-Luis, 2008).	Analysis of variance showed a statistically significant difference when comparing the general score on the attitude scale between the experimental and control groups (*p* = 0.04) after the clinical posting
Esen Danaci *et al*. (2016)	Turkey	106	An hour of theoretical lesson in the third year, as well as watching a documentary and a 3-week psychiatry internship in the fifth year (medical students)	Pre-post	32-item questionnaire designed for rating the attitudes towards schizophrenia, used in different studies in Turkey (binary response format)	Statistically significant change (*p* < 0.001) in percentage of agreement with six out of seven items on social distance.
Fernandez *et al*. (2016)	Malaysia	102	Brief psychoeducation program on reducing stigma in preclinical medical students, consisting of educational lecture and contact intervention (video *v*. face-to-face)	Randomised controlled trial, comparing face-to-face and video-based contact intervention	OMS-HC (Modgill *et al*. 2014)	− Significant main effect of time on OMS-HC total score (*p* < 0.001, partial *η*^*2*^ = 0.49) and subscales (*p* < 0.001, partial *η*^*2*^ between 0.14 and 0.24) – No significant effect of condition on OMS-HC total score and subscales. – Pairwise comparisons revealed significant differences between baseline and follow-up on all subscales in both conditions.
Iheanacho *et al*. (2014)	Nigeria	57	4-day review of basic information on mental illness and psychiatric treatment (nursing and medical students)	Pre-post	Questionnaire based on the World Psychiatric Association Programme to Reduce Stigma and Discrimination because of Schizophrenia, as well as Taylor and Dear (1981) and Wolff *et al*. (1996) (binary response format)	− Statistically significant difference between pre- and post-assessment on three out of four subscales (i.e. socializing with people with mental illness, favourable attitudes toward normal activities and relationships for people with mental illness, and biopsychosoical perspective on the aetiology of mental disorders). – Non-significant difference on the fourth subscale (i.e. belief in witchcraft or curses as cause of mental disorder).
Junqueira *et al*. (2015)	Brazil	120	Brief intervention training for alcohol problems (nursing studies)	Randomised controlled trial	Seaman-Mannello Nurse's Attitudes Toward Alcohol and Alcoholism Scale (Seaman and Mannello, 1978)	Statistically significant change between pre- and post-assessment scores (*p* < 0.001) in both groups.
Keynejad *et al*. (2016)	UK and Somaliland	44	Pairs of students held ten fortnightly meetings to discuss psychiatry topics via the website MedicineAfrica	Pre-post	− ATP-30 (Burra *et al*., 1982) – Stigma questionnaire developed for this study	− More positive ATP-30 scores after the intervention in Somaliland students (*p* = 0.04) but not in UK students (*p* = 0.23). – No significant change on stigma questionnaire.
Rong *et al*. (2011)	China	205	Educational intervention package on ‘better understanding depression’ (medical students)	Two-arm controlled trial comparing two different didactic methods (lecture *v*. lecture + self-directed learning)	MICA (Kassam *et al*., 2010)	− Significant group × time interaction on MICA scores (*p* = 0.007). – Significant decrease of MICA score (indicating a decrease in stigmatising attitudes) within ‘self-directed learning’ group at three follow-ups (*p* = 0.001; *p* = 0.033; and *p* = 0.011, respectively).
Sarikoc *et al*. (2017)	Turkey	86	Interview with standardised patient (nursing students)	Randomised controlled trial	Level of anxiety when conducting an interview with a person with mental illness	Lower level of anxiety at post-assessment in the intervention group (*p* = 0.001).

OMS-HC, Opening Minds Stigma Scale for Health Care Providers; ATP, Attitude to Psychiatry Questionnaire; MICA, Mental Illness: Clinician's Attitude Scale.

### Content and didactic methods

In most studies (*k* = 8), training provided some kind of theoretical background, with only two studies mentioning explicitly that they had addressed the topic of stigma. In five studies, treatment of mental disorders was covered, one study aimed at improving diagnostic skills, and two studies addressed the relationship with patients. Only one study addressed cultural aspects of mental disorders in their training. No study mentioned that they had culturally adapted their training or outcome assessments.

With regard to didactic methods, the vast majority provided lectures (*k* = 8) and two studies used interactive methods. As an example, Rong *et al*. (2011) describe that students had to collect information and organise a half-day advocacy activity about depression in a public place. Two studies used role play and one study used case studies. In three studies, participants underwent a practical training in a psychiatric ward. Of those studies, one reported that participants were exposed to experience with patients, and in another study, participants could attend clinical interviews with patients. Furthermore, seven studies used a contact intervention, in which a patient told their personal story of recovery, either in a video (*k* = 5) or face-to-face (*k* = 2). Outcomes of these interventions were measured using validated questionnaires (*k* = 5) or non-validated questionnaires (*k* = 4). Two studies used a case vignette.

### Outcomes

The descriptive analysis of the main study results is presented in Table 1. One study (Keynejad *et al*., 2016), in which pairs of students in the UK and Somaliland held ten fortnightly meetings to discuss psychiatry topics, showed more positive attitudes towards psychiatry as measured with the Attitude to Psychiatry Questionnaire (ATP-30, Balon *et al*., 1999) at post-assessment, but no significant positive change in the total scores of questionnaires that assessed attitudes towards people with mental illness.

Two studies were mainly based on teaching. In the study by Junqueira *et al*. (2015), nursing students in the experimental group received a brief intervention program for screening, early recognition and the treatment of alcohol problems. The intervention consisted of lectures (12 h) and 4 h in-class practice, whereas the control condition received no intervention. Statistically significant changes in attitudes (*p* < 0.001) were reported for both groups. And in the study by Iheanacho *et al*. (2014), medical and nursing students received a 4-day review of basic information on mental illness and psychiatric treatment. Results showed a statistically significant change in three out of four sub-scales of an attitudinal measure.

Two studies examined the effect of clinical postings, combined with other didactic methods. In the study by Esen Danaci *et al*. (2016), medical students received a theoretical lecture in the third year. Furthermore, they watched a movie and attended interviews with people with schizophrenia during their 3-weeks internship in the fifth year. On six out of seven items of a social distance scale, a statistically significant change in the percentage of agreement was found. de Vargas (2013) compared two groups of nursing students, one who did their 6-weeks practicum in a service that was specialised in abuse of alcohol (*n* = 56) and the other in a non-specialised service (*n* = 144). The authors found a statistically significant group difference at the end of the practicum, with students in the specialised service showing more favourable attitudes towards people affected by abuse of alcohol (*p* = 0.04).

With regard to contact interventions, results were mixed. Altindag *et al*. (2006) examined a contact intervention, in which a young person with schizophrenia was introduced, and a video was shown presenting different people with schizophrenia. The authors reported a statistically significant change in one out of seven items of a social distance questionnaire between pre-assessment and 1-month follow-up. By contrast, Fernandez *et al*. (2016) found more robust results of a contact intervention. They compared a face-to-face with a video-based contact intervention in a randomised controlled trial with medical students. Both groups received an educational lecture before the contact intervention. A significant main effect of time on the total score of the Opening Minds Stigma Scale for Health Care Providers (Modgill *et al*., 2014) emerged (*p* < 0.001, partial *η*^2^ = .49), but no statistically significant difference between the two conditions (video vs. face-to-face) was found. In two other studies, the video-based contact was combined with other interventions (Rong *et al*., 2011; Esen Danaci *et al*., 2016), thus results cannot be attributed to the contact intervention only.

Results generally show a positive effect of engaging students in active tasks. One study delivered an educational intervention package on ‘better understanding depression’ (Rong *et al*., 2011), which included an awareness-raising activity, video-based contact intervention, and group discussions. They found a positive effect of a self-directed learning method (when compared to lectures only) on medical students’ attitudes towards people with mental illness, as measured with the Mental Illness: Clinician's Attitude Scale (Kassam *et al*., 2010). And in the study by Sarikoc *et al*. (2017), nursing students watched a video showing a professional interview with ‘a patient with depression having suicidal ideation’ and ‘a patient with hallucinations’. Thereafter, students in the experimental condition conducted an interview themselves with lay actors who played these scenarios. Results showed that students in the experimental condition felt more competent and reported less anxiety about performing interviews with people with mental health problems than students in the control condition, who had only watched the video.

### Quality of studies

The methodological quality assessment of the included studies (provided in the online supplementary material) showed that the quality of five studies was low. Two studies showed moderate and two showed high methodological quality. Six studies had used a control group, four of which had applied random allocation. Three studies did not report on missing data and two studies showed a high percentage of incomplete data (31% and 60%). None of the studies reported how they handled missing data statistically.

## Discussion

This systematic review examined interventions to reduce mental health-related stigma among medical and nursing students in LMICs. A total of nine studies were included. This number is promising, as a previous systematic review (Yamaguchi *et al*., 2013) identified a lack of studies with medical and nursing students, and when looking at the original studies included in this review, only three had been conducted in LMICs. The studies included in the present review were conducted in six different countries (Brazil, China, Malaysia, Nigeria, Somaliland and Turkey). Despite the promising increase, the number is still small, and there is a clear geographical lack of studies from South America, Central Asia and Arab countries, as has been found in a similar review that focused on medical students’ attitudes towards psychiatry in lower-income countries (Nortje and Seedat, 2013). This result can partly be explained by the fact that no search engines in languages other than English were searched. However, no paper was excluded due to language.

All studies reported significant positive results in at least one of their outcome measures. This result is promising, as well, showing that in general, negative attitudes towards people with mental disorders can be improved with specific interventions among medical and nursing students. From the available literature, it is difficult to draw conclusions on the most effective interventions. No meta-analysis could be calculated due to the small number of studies and the large heterogeneity of outcome measures and evaluation strategies. The methodological quality of the majority of included studies was low, which is likely to affect the reliability of results. Nevertheless, the descriptive analysis of the nine included studies provided valuable insights and directions for future research.

Inconsistent results emerged regarding contact interventions in this review. One study found a significant change in only one out of seven items measuring social distance after a face-to-face contact intervention (Altindag *et al*., 2006). Another study found no statistical difference between a face-to-face and video-based contact intervention (Fernandez *et al*., 2016). In a previous systematic review, Yamaguchi *et al*. (2013) had identified two studies comparing face-to-face *v*. video-based contact, one of which showed that face-to-face had a stronger effect on students’ attitudes and behaviour than video-based contact (Faigin and Stein, 2008), whereas the other found no significant effect (Reinke *et al*., 2004). Thus, more research is needed, and particularly research based on rigorous designs, on how contact interventions are best implemented to show positive effects.

Evidence from anti-stigma interventions with health professionals revealed the following ‘key ingredients’ for effective interventions (Knaak *et al*., 2014): (i) social contact with a trained speaker who has a lived experience of mental illness; (ii) other forms of interventions such as video, where different speakers are featured; (iii) a focus on behavioural change by teaching skills that help health providers know what to do and what to say when working with people with mental illness; (iv) addressing myths; (v) a focus on recovery, demonstrating competence and successful living of people with lived experience of mental illness. Meta-analytic evidence from other fields of research shows that interventions are more effective if they increase empathy and reduce anxiety (Pettigrew and Tropp, 2008).

While some studies included in the present review used some form of social contact or video interventions (the first and second key ingredient suggested by Knaak *et al*., 2014), most studies did not address behavioural change by training specific communication skills to prepare students for their contact with people with mental disorders. Providing theoretical information through lectures was the most frequent intervention, and more practical interventions targeting discriminatory behaviours, such as role plays or clinical practice under supervision, were rarely used. One exception was the study by Sarikoc *et al*. (2017), whose quality was rated as high. In this study, nursing students learned how to conduct interviews with patients (based on a role play with lay actors). After the intervention, students reported feeling more competent and less anxious about contact with real patients than students in the control condition. Anxiety and other unpleasant feeling are one important aspect of how mental health-related stigma is manifested (Thornicroft *et al*., 2007), thus enhancing confidence and reducing anxiety through skills training seems to be an effective way of reducing mental health-related stigma among professional students.

It is highly likely that if medical students feel more confident and acquire better communication skills, they will then have better experiences, and this will lead them to have more positive responses when encountering people with mental illness. The importance of clinical skills and confidence for positive attitudes is supported by findings from primary care workers in high-income and LMICs (Vistorte *et al*., 2018). Health professional students likely need to have a certain level of skills and confidence before their clinical posting, in order to have non-aversive conversations with patients. By contrast, their attitudes might be more negative after an encounter with a patient that was difficult for them. This is highlighted by studies in LMICs which show that student's attitudes may even worsen after their posting in a psychiatric ward (Nortje and Seedat, 2013).

In the present review, the lack of focus on behavioural skills in original studies was also reflected in their outcome measures, which in most cases assessed attitudes only, but not discriminatory behaviour (or behaviour intentions). The same result was found in a previous systematic review on anti-stigma interventions with health professionals in LMICs (Heim *et al*., 2018). Measuring behavioural outcomes is a challenge, particularly in resource-strained contexts, because it requires time and human resources to assess students’ skills in their contact with patients (Nortje and Seedat, 2013). In high-income countries such as the UK, Objective Structured Clinical Examination (OSCE) are used as standard assessments of clinical skills. In OSCEs, communication skills can be observed directly. The use of such an OSCE is described in a protocol for a study in which an anti-stigma intervention will be tested among medical students in several low-, middle-, and high-income countries (Deb *et al*., 2018). In this study, outcomes will be measured with an OSCE that includes communication skills in patient contact, as well as the ability to acknowledge empathetically the impact of stigma when a patient raises this issue.

When training clinical skills and non-discriminatory behaviours among professional students, it is also important to consider the conditions of their clinical rotations and postings in psychiatry wards. A review (Nortje and Seedat, 2013) shows that in many lower-income countries, the conditions for psychiatric training are far from ideal. Psychiatric training is either absent, or it is done in large, psychiatric institutions, where people with severe and chronic forms of mental illness are treated. Students are likely to have negative experiences due to the environment they encounter, the severity of illness they witness, and the negative attitudes they observe in other health personnel, which is also known as the ‘hidden curriculum’ in medical training (Lempp and Seale, 2004). In view of the increasing integration of mental health into primary care in LMICs, it is relevant to find new ways of training medical and nursing students, where they gather experiences with less severe forms of illness, and with patients who recover (Nortje and Seedat, 2013). Such experiences would most probably improve students’ attitudes towards people with mental disorders, and psychiatry as a field of work in general.

And finally, the question arises to what extent insights on effective anti-stigma interventions can be transferred from one cultural group to another. Mental health-related stigma is shaped by culture (Yang *et al*., 2007), and hence there might be cultural differences in the effectiveness of anti-stigma interventions. Among the general public there are differences in what aspects of mental health are stigmatised and how the same stigma assessment tool may be interpreted across cultures and populations (Pescosolido *et al*., 2013; Yang *et al*., 2014). This cultural variation in public attitudes will also be reflected among health professional students. Aside from other factors, it is possible that differences in results of specific interventions (e.g. face-to-face or video-based contact intervention) across studies are caused by cultural factors.

In summary, the results of studies included in this review were mixed, which can partly be explained by the different levels of methodological rigour. Our results suggest that if an intervention is added to a class and does not include own practice, measuring the effects of this intervention in terms of knowledge and attitude will most likely show a short-time effect (e.g. improved attitudes). However, such changes in attitudes might not be long-lasting, and they are merely weak moderators of the ultimately desired outcomes (i.e. changed behaviour). One study showed that even a contact-based intervention had a short-term effect on attitudes (i.e. significant change on three out of seven items on social distance), but 1 month later the difference between control and intervention group in two out of the three items was no longer significant (Altindag *et al*., 2006). Training communication skills, which enhances students’ confidence and reduces their anxiety, as done in a rigorous study by Sarikoc *et al*. (2017), seems to be more promising.

## Limitations

This review has several limitations. First, we relied on previous reviews for the time before 2013. Second, included studies were published in English, and only papers with an English Abstract were included. Full-text screening in other languages was done where necessary and no paper was excluded due to language. But no specific search engines for other languages such as Scielo were used, and we did not include grey literature. Third, we did not look at the outcomes of the interventions in terms of knowledge. Measuring knowledge among medical and nursing students is a separate topic which would have been beyond the scope of the current review, due to its main focus on attitudes and behaviour. And finally, it is most likely that there is a publication bias, with studies that found no effect of their anti-stigma interventions not being published.

## Conclusions

This systematic review adds relevant evidence to the field of interventions to reduce mental health-related stigma among medical and nursing students in LMICs. It shows that in recent years, an increasing number of studies have been published. Results of these studies were generally promising. However, we identified a clear lack of focus on providing training in clinical and communication skills, and measuring behavioural outcomes, which is crucial in interventions to reduce mental health-related stigma. Moreover, there is a lack of implementing and reporting cultural adaptation of anti-stigma interventions, despite the fact that stigma is shaped by culture. For future research and practice, it is of utmost importance to target discriminatory behaviours alongside negative attitudes, in order to enhance the quality of care for people affected by mental disorders worldwide.

## Supplementary material

For supplementary material accompanying this paper visit http://dx.doi.org/10.1017/S2045796019000167. click here to view supplementary material

## Availability of data and materials

The data extraction spreadsheet (Excel) is available on request, please contact the corresponding author.
