# Supplementary material for: Reducing mental health-related stigma among medical and nursing students in low- and middle-income countries: a systematic review
Source: Epidemiol Psychiatr Sci. 2019 Apr 1;29:e28. doi: 10.1017/S2045796019000167 (PMC6848770; doi:10.1017/S2045796019000167)
Supplement: Supplementary file 1 [file S2045796019000167sup001.pdf]

Methodological quality of studies

	Control group	Randomi–zation	Random sequence generation	Drop-out below 5%	Quality of the study
Altindag et al. (2006)	yes	no	n.a.	uncertain	low
de Vargas et al. (2013)	yes	yes	uncertain	yes	moderate
Esen Danaci et al. (2016)	no	n.a.	n.a.	uncertain	low
Fernandez et al. (2016)	yes	yes	yes	yes	high
Iheanacho et al. (2014)	no	n.a.	n.a.	no	low
Junqueira et al. (2015)	yes	yes	uncertain	yes	moderate
Keynejad et al. (2016)	no	n.a.	n.a.	no	low
Rong et al. (2011)	yes	no	n.a.	uncertain	low
Sarikoc et al. (2017)	yes	yes	yes	yes	high

Table 1. Intervention content

Reference	Theory	Stigma as a topic	Diagnostic skills	Treatment /interventions /practices	Relationship with patients	Cultural aspects
Altindag *et al.* (2006)	X	X		X		
de Vargas (2013)	X					
Esen Danaci *et al.* (2016)	X					
Fernandez *et al.* (2016)	X	X				
Iheanacho *et al.* (2014)	X			X	X	
Junqueira *et al.* (2015)	X		X	X		
Keynejad *et al.* (2016)						X
Rong *et al.* (2011)	X			X	X	
Sarikoc *et al.* (2017)	X			X		

Table 2. Didactic methods

Reference	Lecture	Interactive class	Case study	Role play	Contact (video)	Contact (in person)	Clinical posting	Own practise	Attend interviews
Altindag *et al.* (2006)	X				X	X			
de Vargas (2013)	X						X		
Esen Danaci *et al.* (2016)	X				X		X		X

Reference	Lecture	Interactive class	Case study	Role play	Contact (video)	Contact (in person)	Clinical posting	Own practise	Attend interviews
Fernandez *et al.* (2016)	X				X	X			
Iheanacho *et al.* (2014)	X	X		X					
Junqueira *et al.* (2015)	X	X							
Keynejad *et al.* (2016)		X							
Rong *et al.* (2011)	X	X			X				
Sarikoc *et al.* (2017)	X			X					

Table 3. Mental disorders addressed and cultural adaptation of the intervention

Reference	Depression	Alcohol	Schizophrenia	Mental disorders in general	Cultural adaptation
Altindag *et al.* (2006)			X		
de Vargas (2013)		X			
Esen Danaci *et al.* (2016)			X		
Fernandez *et al.* (2016)				X	
Iheanacho *et al.* (2014)				X	
Junqueira *et al.* (2015)		X			
Keynejad *et al.* (2016)				X	

Reference	Depression	Alcohol	Schizophrenia	Mental disorders in general	Cultural adaptation
Rong *et al.* (2011)	X				X
Sarikoc *et al.* (2017)	X		X		

MEDLINE SEARCH STRATEGY
2017-05-06

1. Stigma (Mehta et al., 2015; Thornicroft et al., 2016)
Stereotyping/ OR Social Stigma/ OR Prejudice/ OR Attitude/ OR Social Perception/ OR "Attitude of Health Personnel"/ OR Social Distance/ OR "Rejection (Psychology)"/ OR Human Rights/ OR Civil Rights/ OR Social Justice/ OR "Discrimination (Psychology) "/ OR Social discrimination/ OR human rights OR civil rights OR social justice OR discrimination OR injustice OR stigma* OR stereotyp* OR social distanc* OR marginali*

Additional terms by Henderson et al. (2014): prejudi* OR Ignoran* OR Misconception*1 OR Misperception*1 OR hostil* OR emotional reaction OR disrespect* OR victimi* OR abusive OR patronis* OR patroniz* OR misdiagnos* OR under treatment OR non treatment OR no access OR less access OR diagnostic overshadow* OR attitud* OR behavior*1 OR behaviour*1 OR opinion*1 OR view* OR assum* OR judg*

Additional terms by authors: Professional Competence/ OR Quality of Health Care/ OR Patient Satisfaction/ OR Patient Acceptance of Health Care/ OR Burnout, Professional/ OR case detection OR competen* OR practice OR skills OR burnout OR dignity OR help-seeking

Exclude (from Mehta et al., 2015; Thornicroft et al., 2016)
6. Interpersonal Relations/
7. public opinion/
14. public opinion
19. empower*

2. Mental health (Mehta et al., 2015; Thornicroft et al., 2016)
Mental Health/ OR Mental Disorders/ OR Mentally Ill Persons/ OR Somatoform Disorders/ OR Depression/ OR Depression, Postpartum/ OR common mental disorders OR Depressive Disorder/ OR Depressive Disorder, Major/ OR Psychotic Disorders/ or Affective Disorders, Psychotic/ OR Neurotic Disorders/ or Self-Injurious Behavior/ OR Anxiety/ OR Anxiety Disorders/ OR Schizophrenia, Paranoid/ OR Schizophrenia/ OR Bipolar Disorder/ OR Mood Disorders/ OR Obsessive-Compulsive Disorder/ OR Personality Disorders/ OR Eating Disorders/ OR Anorexia Nervosa/ OR Anorexia/ OR Bulimia Nervosa/ or Bulimia/ OR Mental Health Services/ OR mental health OR mental illness OR psychotic OR psychosis OR schizo* OR bipolar disorder OR manic depression OR OCD OR obsessive compulsive disorder OR anorex* OR bulimi*

Additional terms by Henderson et al. (2014): post-traumatic stress OR alcohol* OR substance OR illicit drugs

Additional terms by authors: OR PTSD OR distress

3. Intervention (Mehta et al., 2015; Thornicroft et al., 2016)
randomized controlled trial.pt. OR controlled clinical trial.pt. OR evaluation studies.pt. OR Prospective Studies/ OR Follow-Up Studies/ OR Social Marketing/ OR National Health Programs/ OR Government Programs/ OR Program Development/ OR Learning/ or Multimedia/ or Teaching/ OR Internet/ OR Video-Audio Media/ OR Video Recording/ OR Software/ OR Persuasive Communication/ OR Health Knowledge, Attitudes, Practice/ OR Awareness/ OR Patient Advocacy/ OR Health Promotion/ OR Intervention Studies/ OR Randomized Controlled Trials as Topic/

OR evaluat* OR intervention* OR randomi* OR follow-up OR pre-post OR time series OR time point OR message OR marketing OR program* OR newsletter* OR DVD OR website OR compact disc OR stigma resilience OR stigma change OR (stigma* adj5 cop*) OR (stigma* adj5 reduc*) OR

(discrim* adj5 reduc*) OR (stigma* adj5 challeng*) OR (discrim* adj5 challeng*) OR (stigma* adj5 combat*) OR (discrim* adj5 combat*) OR (stigma* adj5 counter*) OR (discrim* adj5 counter*) OR contact OR testimon* OR mental health literacy OR training

(excluded)
66. Mass Media/
69. Journalism
70. Video Games/
72. Pamphlets/
73. Advertising as Topic/
74. Public Relations/
75. Public Relations/
77. Famous Persons/
80. Consumer Advocacy/
91. campaign
93. advert*
95. public relation*
97. newspaper*
98. magazine*
100. broadcast
101. radio
102. television
103. cinema
107. movie
108. film
109. trailer

4. Health care (Henderson et al., 2014)

health* professional OR health* personnel OR Doctor*1 OR psychiat* OR clinical psych* OR nurs* OR occupational therap* OR medical student*1 OR health* worker*1 OR paramedic* OR ambulance service OR mental health social worker*1 OR Medic*1 OR Clinician*1 OR Physician*1 OR case manager*1 OR key worker*1 OR keyworker*1 OR psychotherapist*1 OR hospital* OR a&e OR Emergency department*1 OR Health* provider*1 OR Health* staff OR general practitioner*1

Additional terms by authors: Health personnel/ OR Professional-Patient Relations/ OR psychology students

(excluded)
midwi*
physiotherap*
Pharmacist*1
Pharmacy student*1
dietitian*1
dietician*1
Dentist*1
Surgeon*1
Obstetrician*1
Dental hygienist*1
Gynaecologist*

5. LMIC country list

Afghanistan.mp OR Albania.mp OR Algeria.mp OR American Samoa.mp OR Angola.mp OR Argentina.mp OR Armenia.mp OR Azerbaijan.mp OR Bangladesh.mp OR Belarus.mp OR Belize.mp OR Benin.mp OR Bhutan.mp OR Bolivia.mp OR Bosnia OR Herzegovina OR Botswana.mp OR Brazil.mp OR Bulgaria.mp OR "Burkina Faso".mp OR Burundi.mp OR "Cape Verde".mp OR Cambodia.mp OR Cameroon.mp OR "Central African Republic".mp OR Chad.mp OR China.mp OR Colombia.mp OR Comoros.mp OR Congo.mp OR Costa Rica.mp OR "Cote d'Ivoire".mp OR Cuba.mp OR Djibouti.mp OR Dominica.mp OR Dominican Republic.mp OR Ecuador.mp OR Egypt.mp OR "El Salvador".mp OR "Equatorial Guinea".mp OR Eritrea.mp OR Ethiopia.mp OR Fiji.mp OR Gabon.mp OR Gambia.mp OR Georgia.mp OR Ghana.mp OR Grenada.mp OR Guatemala.mp OR Guinea.mp OR Guinea-Bissau.mp OR Guyana.mp OR Haiti.mp OR Honduras.mp OR India.mp OR Indonesia.mp OR Iran.mp OR Iraq.mp OR Jamaica.mp OR Jordan.mp OR Kazakhstan.mp OR Kenya.mp OR Kiribati.mp OR Korea.mp OR Kosovo.mp OR Kyrgyzstan.mp OR Laos.mp OR Lebanon.mp OR Lesotho.mp OR Liberia.mp OR Libya.mp OR Macedonia.mp OR Madagascar.mp OR Malawi.mp OR Malaysia.mp OR Maldives.mp OR Mali.mp OR "Marshall Islands".mp. OR Mauritania.mp OR Mauritius.mp OR Mexico.mp OR Micronesia.mp OR Moldova.mp OR Mongolia.mp OR Montenegro.mp OR Morocco.mp OR Mozambique.mp OR Myanmar.mp OR Namibia.mp OR Nepal.mp OR Nicaragua.mp OR Niger.mp OR Nigeria.mp OR Pakistan.mp OR Palau.mp OR Panama.mp OR "Papua New Guinea".mp OR Paraguay.mp OR Peru.mp OR Philippines.mp OR Romania.mp OR Russia.mp OR Rwanda.mp OR Samoa.mp OR "Sao Tome and Principe".mp OR Senegal.mp OR Serbia.mp OR "Sierra Leone".mp OR Melanesia.mp OR Somalia.mp OR "South Africa".mp OR "South Sudan".mp OR "Sri Lanka".mp OR "Saint Lucia".mp OR "Saint Vincent and the Grenadines".mp OR Sudan.mp OR Suriname.mp OR Swaziland.mp OR Syria.mp OR Tajikistan.mp OR Tanzania.mp OR Thailand.mp OR Timor-Leste.mp OR Togo.mp OR Tonga.mp OR Tunisia.mp OR Turkey.mp OR Turkmenistan.mp OR Tuvalu.mp OR Uganda.mp OR Ukraine.mp OR Uzbekistan.mp OR Vanuatu.mp OR Venezuela.mp OR Vietnam.mp OR "West Bank".mp OR Gaza.mp OR Palestine.mp OR Yemen.mp OR Zambia.mp OR Zimbabwe.mp

Afghanistan/ OR Albania/ OR Algeria/ OR American Samoa/ OR Angola/ OR Argentina/ OR Armenia/ OR Azerbaijan/ OR Bangladesh/ OR Belarus/ OR Belize/ OR Benin/ OR Bhutan/ OR Bolivia/ OR "Bosnia and Herzegovina"/ OR Botswana/ OR Brazil/ OR Bulgaria/ OR "Burkina Faso"/ OR Burundi/ OR Cape Verde/ OR Cambodia/ OR Cameroon/ OR Central African Republic/ OR Chad/ OR China/ OR Colombia/ OR Comoros/ OR Congo/ OR Costa Rica/ OR Cote d'Ivoire/ OR Cuba/ OR Djibouti/ OR Dominica/ OR Dominican Republic/ OR Ecuador/ OR Egypt/ OR El Salvador/ OR Equatorial Guinea/ OR Eritrea/ OR Ethiopia/ OR Fiji/ OR Gabon/ OR Gambia/ OR Georgia/ OR Ghana/ OR Grenada/ OR Guatemala/ OR Guinea/ OR Guinea-Bissau/ OR Guyana/ OR Haiti/ OR Honduras/ OR India/ OR Indonesia/ OR Iran/ OR Iraq/ OR Jamaica/ OR Jordan/ OR Kazakhstan/ OR Kenya/ OR Kiribati/ OR "Democratic People's Republic of Korea"/ OR Kosovo/ OR Kyrgyzstan/ OR Laos/ OR Lebanon/ OR Lesotho/ OR Liberia/ OR Libya/ OR "Macedonia (Republic)"/ OR Madagascar/ OR Malawi/ OR Malaysia/ OR Maldives/ OR Mali/ OR Marshall Islands.mp. OR Mauritania/ OR Mauritius/ OR Mexico/ OR Micronesia/ OR Moldova/ OR Mongolia/ OR Montenegro/ OR Morocco/ OR Mozambique/ OR Myanmar/ OR Namibia/ OR Nepal/ OR Nicaragua/ OR Niger/ OR Nigeria/ OR Pakistan/ OR Palau/ OR Panama/ OR Papua New Guinea/ OR Paraguay/ OR Peru/ OR Philippines/ OR Romania/ OR Russia/ OR Rwanda/ OR Samoa/ OR (Sao Tome and Principe).mp OR Senegal/ OR Serbia/ OR Sierra Leone/ OR Melanesia/ OR Somalia/ OR South Africa/ OR South Sudan/ OR Sri Lanka/ OR Saint Lucia/ OR "Saint Vincent and the Grenadines"/ OR Sudan/ OR Suriname/ OR Swaziland/ OR Syria/ OR Tajikistan/ OR Tanzania/ OR Thailand/ OR Timor-Leste/ OR Togo/ OR Tonga/ OR Tunisia/ OR Turkey/ OR Turkmenistan/ OR Tuvalu.mp OR Uganda/ OR Ukraine/ OR Uzbekistan/ OR Vanuatu/ OR Venezuela/ OR Vietnam/ OR West Bank.mp OR Gaza.mp OR Palestine.mp OR Yemen/ OR Zambia/ OR Zimbabwe/

[retrieved from http://data.worldbank.org/income-level/low-and-middle-income on 24 March 2017]

References

Henderson, C., Noblett, J., Parke, H., Clement, S., Caffrey, A., Gale-Grant, O., . . . Thornicroft, G. (2014). Mental health-related stigma in health care and mental health-care settings. *The Lancet Psychiatry, 1*(6), 467-482. doi:10.1016/S2215-0366(14)00023-6

Mehta, N., Clement, S., Marcus, E., Stona, A. C., Bezborodovs, N., Evans-Lacko, S., . . . Thornicroft, G. (2015). Evidence for effective interventions to reduce mental health-related stigma and discrimination in the medium and long term: Systematic review. *British Journal of Psychiatry, 207*(5), 377-384. doi:10.1192/bjp.bp.114.151944

Thornicroft, G., Mehta, N., Clement, S., Evans-Lacko, S., Doherty, M., Rose, D., . . . Henderson, C. (2016). Evidence for effective interventions to reduce mental-health-related stigma and discrimination. *The Lancet, 387*(10023), 1123-1132. doi:http://dx.doi.org/10.1016/S0140-6736(15)00298-6
